# Emotional blind spots and dark minds: how alexithymia and mindfulness shape the link between Dark Triad personality traits and psychopathology

**DOI:** 10.3389/fpsyg.2025.1677305

**Published:** 2025-11-21

**Authors:** Angelika Ecker, David A. Preece, Daniel Schleicher, Ricarda Jacob, Stephanie Kandsperger, Romuald Brunner, Irina Jarvers

**Affiliations:** 1 Department of Child and Adolescent Psychiatry and Psychotherapy, University of Regensburg, Regensburg, Germany; 2 School of Population Health, Curtin University, Perth, WA, Australia; 3 Department of Psychology, Stanford University, Stanford, CA, United States

**Keywords:** Dark Triad, psychopathology, depression, anxiety, stress, alexithymia, mindfulness, moderation analysis

## Abstract

**Introduction:**

Dark Triad traits (narcissism, Machiavellianism, psychopathy) are linked to increased psychological distress, including depression, anxiety, and stress. While the underlying mechanisms remain unclear, alexithymia has emerged as a potential risk factor, and mindfulness as a possible resilience factor. This study explores the associations and moderating effects of alexithymia and mindfulness on psychopathology within the Dark Triad spectrum.

**Methods:**

A total of 577 university students completed a cross-sectional online survey (age range 16–50 years, *M* = 22.73, *SD* = 6.19, 75.2% female) comprising several questionnaires. Dark Triad traits were assessed using the Dirty Dozen (DD), psychopathology was measured with the Depression Anxiety Stress Scales (DASS-21), alexithymia with the Perth Alexithymia Questionnaire (PAQ), and mindfulness with the Five Facet Mindfulness Questionnaire (FFMQ).

**Results:**

Kendall’s tau-b correlations revealed positive associations between all three Dark Triad traits and facets of psychopathology [*r*(0.12; 0.18), all *p* < 0.001]. Regression analyses confirmed specific associations with psychopathology, even when controlling for alexithymia and mindfulness. Moderation analyses were then conducted for significant Dark Triad predictors, focusing on alexithymia and mindfulness. The association between Machiavellianism and both depression and anxiety was moderated by alexithymia (= difficulties appraising negative feelings, *p* < 0.05), and additionally by mindfulness (= non-judging attitude, *p* < 0.01) for depression, showing nuanced and complex interaction patterns. Alexithymia regarding negative emotions moderated the psychopathy–stress link (*p* < 0.05).

**Discussion:**

Small but consistent associations emerged between Dark Triad traits and psychopathology, which were better understood when accounting for gender, alexithymia, and mindfulness. Machiavellianism showed complex, non-linear moderation effects. In contrast, alexithymia regarding negative feelings consistently moderated the link between stress and psychopathy, while narcissism showed no significant associations after controlling for influencing variables.

**Conclusion:**

Dark personality traits are linked to psychological vulnerability, with particularly complex patterns observed for Machiavellianism. Depending on individual levels of alexithymia and mindfulness, these constructs may act as either risk or protective factors. These findings help to uncover the nature of the links between the Dark Triad and psychopathology and reveal novel intervention targets.

## 1 Introduction

The Dark Triad refers to three interrelated but distinct personality traits: psychopathy, Machiavellianism, and narcissism (Paulhus and Williams, 2002). Psychopathy is characterized by impulsivity, low empathy, emotional detachment, fearlessness, and antisocial behavior, with distinctions made between primary (low anxiety, high emotional detachment) and secondary psychopathy (high impulsivity and emotionality) (Jones and Paulhus, 2014; Newman et al., 2005). Machiavellianism involves manipulativeness, a cynical worldview, and emotional detachment, often expressed through strategic planning and a lack of moral concern (Christie and Geis, 2013; Jones and Paulhus, 2014). Narcissism is defined by grandiosity, a constant need for admiration, and exploitative interpersonal behavior (Paulhus and Williams, 2002; Jakobwitz and Egan, 2006). While these traits share features such as manipulativeness and low empathy, they differ in impulsivity and interpersonal strategy—psychopathy being more reckless, Machiavellianism more calculated, and narcissism more self-centered (Jones and Paulhus, 2014; Hare and Neumann, 2008). Subclinical forms of these Dark Triad traits exist along a continuum in the general population and can, in some contexts, confer advantages—particularly in professional settings (Jakobwitz and Egan, 2006). A recent meta-analysis by Blasco-Belled et al. (2024) examined well-being in the context of the Dark Triad traits: grandiosity in narcissism and boldness/dominance in psychopathy were associated with higher levels of well-being, whereas other patterns—such as vulnerable narcissism, antagonism, and disinhibition in psychopathy, and Machiavellianism—were linked to lower levels of well-being. These findings suggest that the frequent experience of negative emotions within certain Dark Triad traits can have detrimental effects on well-being (Blasco-Belled et al., 2024). Building on these findings, it is important to recognize that well-being represents only one dimension of mental health. To gain a more comprehensive understanding, it is equally crucial to examine ill-being, which encompasses a range of psychopathological symptoms. These symptoms offer additional insights and reflect the complexity of how different Dark Triad traits and their specific facets relate to mental health.

The relationship between Dark Triad traits and psychopathology appears to be complex and varies across traits and their facets. Within narcissism, two distinct subtypes can be differentiated: grandiose narcissism is characterized by extraversion, self-assurance, exhibitionism, and aggression, whereas vulnerable narcissism involves introversion, defensiveness, anxiety, and vulnerability to life’s traumas (Wink, 1991). Notably, the potentially adaptive aspect of grandiose narcissism is reflected in lower reported severity of symptoms such as anxiety, depression, and stress (Papageorgiou et al., 2023; Bonfá-Araujo et al., 2023; Gómez-Leal et al., 2019). However, this pattern does not extend to vulnerable narcissism, which has been linked to increased psychological distress. In contrast, Machiavellianism and psychopathy are generally associated with greater vulnerability, showing a positive, albeit weak, correlation with psychopathological symptoms (Papageorgiou et al., 2023; Bonfá-Araujo et al., 2023; Gómez-Leal et al., 2019; Lyons et al., 2019; Birkás et al., 2016). These differential patterns align with findings on overall well-being, where grandiose narcissism may relate to higher well-being, while Machiavellianism and psychopathy tend to correspond with lower levels of well-being. These observations from cross-sectional studies are further supported by a recent longitudinal investigation by Wei and colleagues (Wei et al., 2025). The study found a two-way link between narcissism and psychopathological symptoms: higher narcissism predicted fewer symptoms over time, while more severe symptoms led to lower narcissism. Increases in psychopathy and Machiavellianism were tied to higher psychological symptom levels within individuals. Across individuals, all Dark Triad traits were consistently associated with more psychopathological symptoms (Wei et al., 2025).

Given the complex associations between Dark Triad traits and psychopathology, it is important to consider potential moderating factors that might help explain or influence these relationships. One such factor is alexithymia, a trait involving difficulties in identifying (DIF) and describing (DDF) one’s own emotions, as well as an Externally Oriented Thinking (EOT) style whereby one rarely focuses attention on their emotions (Preece et al., 2017). That is, people high in alexithymia have difficulty at the attention (i.e., EOT) and appraisal (i.e., DIF and DDF) stages of emotion processing. Alexithymia has been consistently identified as a transdiagnostic risk factor for psychopathology, including in adults (Kick et al., 2024; Preece et al., 2022) and in adolescents (Weissman et al., 2020). Several studies have explored how this emotional risk factor interacts with specific Dark Triad traits. Findings consistently show a positive link between Machiavellianism and alexithymia: Machiavellianism is strongly linked to EOT (Jonason and Krause, 2013; Wastell and Booth, 2003; Sheikhi et al., 2017) and shows varying positive correlations with DIF (Wastell and Booth, 2003) and DDF (Sheikhi et al., 2017). These findings suggest that Machiavellian traits are closely related to reduced emotional awareness and a cognitive focus away from internal emotional states. The relationship between narcissism and alexithymia shows mixed patterns: while one study specifically found a positive correlation with DIF (Jonason and Krause, 2013), others reported positive associations with overall alexithymia or all of its subcomponents (Schimmenti et al., 2019; Di Tella et al., 2024). In contrast, one study observed negative associations with EOT and DDF, but no significant link with DIF (Cairncross et al., 2013). A recent meta-analysis found a positive association between alexithymia and psychopathy, suggesting that difficulties in emotional awareness may be a common feature of psychopathic traits (Burghart and Mier, 2022). Overall, these findings highlight alexithymia as a relevant emotional factor in the context of Dark Triad traits, with more consistent associations for Machiavellianism and psychopathy than for narcissism. Given this variability, one possibility is that alexithymia may moderate the relationship between Dark Triad traits and psychopathological outcomes: when individuals experience difficulties in identifying and expressing their emotions, the negative psychological effects associated with Dark Triad traits may be amplified. Specifically, deficits in emotional awareness may limit individuals’ capacity to recognize and respond appropriately to their emotional states, thereby intensifying the maladaptive interpersonal and affective tendencies characteristic of the Dark Triad (e.g., callousness, emotional detachment, or antagonism).

While much research has focused on risk factors associated with Dark Triad traits, it is equally important to consider protective factors that support positive mental health. According to the dual-factor model of mental health, psychological well-being is best understood as comprising both the presence of positive mental health and the absence of mental illness (Greenspoon and Saklofske, 2001). In this context, mindfulness emerges as a key resilience factor that not only protects general mental health but also shows specific relevance in relation to Dark Triad traits. Mindfulness is defined by two core components: the self-regulation of attention directed at present-moment experiences, and an open, non-judgmental acceptance of all internal and external events (Bishop et al., 2004). Recognized as a resilience factor for mental health (Zhang et al., 2023), mindfulness may also exert a buffering effect against the detrimental impact of alexithymia. This assumption is supported by findings from a study with a student sample, in which the alexithymia subscales DIF and DDF were found to be negatively associated with mindfulness dimensions such as quality of mindfulness, awareness, and acceptance (Teixeira and Pereira, 2015). Moreover, mindfulness-based interventions have been shown to reduce alexithymia (Norman et al., 2019).

Beyond its general protective role, mindfulness also appears to show varied effects in relation to Dark Triad traits. Previous research investigating the relationship between Dark Triad traits and the tendency to be mindful (trait mindfulness) has yielded somewhat inconsistent findings. For instance, a non–peer-reviewed student thesis (Scavone, 2017) identified associations between mindfulness and two of the three Dark Triad facets—reporting a weak positive correlation with narcissism and a weak negative correlation with Machiavellianism, while no significant correlation was found with psychopathy. This study was conducted on a sample of nearly 250 university students, 81% of whom were female. Interestingly, this study suggested that alexithymia may mediate the relationship between narcissism and mindfulness, indicating a possible underlying mechanism that warrants further investigation (Scavone, 2017). In contrast, another recent study, which employed a larger and more gender-balanced student sample, found only a weak positive association between psychopathy and mindfulness, with no significant relationships observed for narcissism or Machiavellianism (Aldbyani and Al-Abyadh, 2022). Taken together, these findings highlight the inconsistency of current evidence, suggesting that the relationship between mindfulness and the Dark Triad traits remains unclear and may depend on sample characteristics or methodological differences. Moving beyond these direct associations, a growing body of research has begun to explore mindfulness as a moderating variable in the context of the Dark Triad. For example, Bronchain et al. (2019) demonstrated that mindfulness can attenuate the effect of psychopathy on antisocial behavior. Similarly, a study by Aldbyani and Al-Abyadh (2022) were also able to show that mindfulness may moderate the relationship between Dark Triad traits and mental health outcomes. Although some initial studies have explored mindfulness in relation to the Dark Triad, the existing evidence is limited and lacks consistency across findings. Combining alexithymia and mindfulness in this context offers a novel perspective, as it allows for examining both emotional vulnerability and emotional resilience within individuals exhibiting Dark Triad traits—two mechanisms that may interact in shaping psychological outcomes.

Taken together, while Dark Triad traits have been predominantly linked to adverse psychological outcomes, the mechanisms underlying these associations remain insufficiently understood. In particular, research exploring the joint and potentially interactive effects of alexithymia and mindfulness on the psychological consequences of Dark Triad traits is still scarce. Given the mixed evidence on direct associations, examining alexithymia and mindfulness jointly may help to uncover compensatory or buffering mechanisms that shape the link between Dark Triad traits and psychopathological symptoms. Specifically, mindfulness is considered a potential resilience factor that may buffer the impact of Dark Triad traits on psychological outcomes, whereas alexithymia is treated as a vulnerability factor that may exacerbate these associations by reducing emotional awareness and adaptive coping. Therefore, the present study aims to investigate how these two constructs—alexithymia as a potential vulnerability factor and mindfulness as a possible resilience factor—separately influence the relationship between Dark Triad traits and indicators of psychopathology, providing a clearer understanding of the mechanisms of vulnerability and resilience.

## 2 Methods

### 2.1 Design

This study employed a cross-sectional, between-subjects design based on an online survey conducted with university students. Participants completed a battery of self-report questionnaires as part of a broader ongoing project on emotions and mental health. This study was approved by the Curtin University Human Research Ethics Committee. All participants provided their informed consent prior to participation. To be eligible to participate, participants needed to be undergraduate students currently enrolled in a psychology unit (course) at Curtin University. Participants received course credit for their participation.

### 2.2 Sample

Data were collected between May 2022 and August 2023 at Curtin University in Australia.^1^ The final sample consisted of 577 participants who completed the survey with all the required measures. They had a mean age of 22.73 years (*SD* = 6.19) and 75.2% identified as female. A total of 39.9% reported a current or past diagnosis of a mental disorder, and nearly 50% were engaged in casual or part-time employment. For detailed demographic and clinical characteristics, see Table 1. Some additional participants also completed all the measures for this study, but their data were excluded as they failed a validity check question (*n* = 49).

**Table 1:** Descriptive statistics.

Participant characteristics	
Age in years	
*M* (*SD*)	22.73 (6.19)
Range	16–50
Gender	*n* (%)
Female	434 (75.20)
Male	128 (22.20)
Non-binary/gender diverse	15 (2.60)
Employment status	*n* (%)
Full time	49 (8.50)
Part time	158 (27.40)
Casual	282 (48.90)
Unemployed	88 (15.30)
Diagnosed mental disorder	*n* (%)
No	347 (60.10)
Yes	230 (39.90)
Depression	130 (22.50)
Anxiety	162 (28.10)
Bipolar disorder	9 (1.60)
ADHD	28 (4.90)
Autism	4 (0.70)
Eating disorder	21 (3.60)
PTSD	27 (4.70)
Personality disorder	9 (1.60)
OCD	12 (2.10)
Psychosis	2 (0.30)

Questionnaire measures				
	*M (SD)*	Participants range	Questionnaire range	Cronbach’s alpha
Machiavellianism (DD)	2.85 (1.16)	1.00–6.25	1.00–7.00	0.77
Narcissism (DD)	2.60 (1.07)	1.00–7.00	1.00–7.00	0.78
Psychopathy (DD)	2.81 (1.30)	1.00–7.00	1.00–7.00	0.69
Depression (DASS-21)	7.51 (5.61)	0.00–21.00	0.00–21.00	0.88
Anxiety (DASS-21)	6.66 (5.15)	0.00–21.00	0.00–21.00	0.82
Stress (DASS-21)	8.67 (5.05)	0.00–21.00	0.00–21.00	0.90
General Externally Oriented Thinking (PAQ)	26.76 (10.98)	8.00–56.00	8.00–56.00	0.90
General Difficulty Appraising Feelings (PAQ)	49.13 (20.39)	16.00–111.00	16.00–112.00	0.95
Total Score Alexithymia (PAQ)	75.88 (30.46)	24.00–167.00	24.00–168.00	0.95
Acting with awareness (FFMQ)	2.92 (0.76)	1.00–5.00	1.00–5.00	0.87
Non-judging of inner experience (FFMQ)	3.16 (0.98)	1.00–5.00	1.00–5.00	0.87
Non-reactivity to inner experience (FFMQ)	2.96 (0.82)	1.00–5.00	1.00–5.00	0.75

Patients were able to report multiple diagnoses. ADHD, Attention-Deficit/Hyperactivity Disorder; PTSD, Post-Traumatic Stress Disorder; OCD, Obsessive-Compulsive Disorder; DD, Dirty Dozen; DASS-21, Depression Anxiety Stress Scales; FFMQ, Five Facet Mindfulness Questionnaire; PAQ, Perth Alexithymia Questionnaire.

### 2.3 Measures

#### 2.3.1 Dark Triad

Dark Triad traits were assessed using the Dirty Dozen questionnaire [DD, (Jonason and Webster, 2010)]. The DD is a brief self-report measure consisting of 12 items evenly divided into three subscales assessing *Machiavellianism*, *narcissism*, and *psychopathy*. The narcissism subscale reflects the grandiose rather than the vulnerable form of narcissism. Items are rated on a 7-point Likert scale ranging from 1 (*strongly disagree*) to 7 (*strongly agree*), with higher scores indicating greater expression of the respective trait. Subscale scores are computed by averaging the responses for the four items per trait. The Dirty Dozen has demonstrated acceptable to good internal consistency and its three-factor structure has been supported in multiple validation studies (Jonason and Webster, 2010; Miller et al., 2012).

#### 2.3.2 Psychopathology

Psychopathology was assessed using the Depression Anxiety Stress Scales-21 [DASS-21; (Lovibond and Lovibond, 1995)]. The DASS-21 is a self-report instrument comprising 21 items, which are equally distributed across three subscales: *depression*, *anxiety*, and *stress*. Items are rated on a 4-point Likert scale ranging from 0 (*did not apply to me at all*) to 3 (*applied to me very much or most of the time*), referring to the past week. Higher scores indicate greater symptom severity in the respective domain. The DASS-21 has demonstrated solid psychometric properties, with confirmed three-factor structure and good to excellent internal consistencies (Henry and Crawford, 2005).

#### 2.3.3 Alexithymia

To assess alexithymia, the Perth Alexithymia Questionnaire [PAQ; (Preece et al., 2018)] was administered. The PAQ comprises 24 items rated on a 7-point Likert scale (1 = *strongly disagree*, 7 = *strongly agree*), with higher scores reflecting greater levels of alexithymia. It includes five subscales that distinguish between difficulties in identifying and describing both negative and positive emotions: *Negative-Difficulty Identifying Feelings* (N-DIF), *Positive-Difficulty Identifying Feelings* (P-DIF), *Negative-Difficulty Describing Feelings* (N-DDF), *Positive-Difficulty Describing Feelings* (P-DDF), and *Externally Oriented Thinking* (EOT). These subscales may also be combined into various theoretically meaningful composite scores, such as general DIF, general DDF, or the broader Difficulty Appraising Feelings (DAF) index, which combines all DIF and DDF items and aligns conceptually with the appraisal stage of emotion processing. This can be contrasted with EOT, which reflects the attention stage of emotion processing (Preece and Sikka, 2024). An overall alexithymia score can be obtained by summing all items. In the present study, we focused primarily on the N-DAF, P-DAF, and EOT scores to highlight distinctions between the attention and appraisal stages of emotion processing, as well as between the appraisal of negative versus positive emotions. The PAQ has demonstrated excellent internal consistency in prior research (Preece et al., 2018).

#### 2.3.4 Mindfulness

Mindfulness was measured using the Five Facet Mindfulness Questionnaire (FFMQ), a self-report instrument assessing five facets of trait mindfulness: *observing, describing, acting with awareness, non-judging of inner experience, and non-reactivity to inner experience* (Baer et al., 2006). Specifically, *observing* refers to the tendency to notice internal and external experiences, *describing* reflects the ability to label such experiences with words, and *acting with awareness* involves focusing attention on present-moment activities rather than behaving automatically. *Non-judging of inner experience* denotes an accepting and non-evaluative stance toward one’s thoughts and emotions, while *non-reactivity to inner experience* captures the ability to let thoughts and feelings arise and pass without becoming entangled in them. The FFMQ consists of 39 items, which are rated on a 5-point Likert scale ranging from 1 (*never or very rarely true*) to 5 (*very often or always true*), with several items being reverse-scored. For each facet, a sum score is calculated. Due to the conceptual overlap between the facets *observing* and *describing* and the construct of alexithymia, only the three scales *acting with awareness, non-judging of inner experience*, and *non-reactivity to inner experience* were included in the analyses. The five-factor structure of the FFMQ has been confirmed through both exploratory and confirmatory factor analyses, and the subscales demonstrate good internal consistencies (Baer et al., 2006).

### 2.4 Statistical analysis

All statistical analyses were conducted using IBM SPSS Statistics (Version 29, IBM Corp., Armonk, NY) and R Version 4.4.1 (R Core Team, 2021). Due to violations of normality assumptions, non-parametric methods were employed for all correlational and group comparison analyses. The analysis proceeded in three steps. First, associations between control variables (gender, age) and the primary study variables Dark Triad traits, psychopathology, and mindfulness were examined using Mann–Whitney *U* tests and Kendall’s *τ*-*b* correlations. Second, Kendall’s *τ*-*b* correlations were computed to assess bivariate relationships between the three Dark Triad traits (Machiavellianism, narcissism, psychopathy), indicators of psychopathology (depression, anxiety, stress), mindfulness facets (acting with awareness, non-judging of inner experience, non-reactivity to inner experience) and alexithymia (N-DAF, P-DAF, EOT). Third, three multiple linear regression models were conducted with depression, anxiety, and stress as outcome variables. Before conducting these analyses, the assumptions of linear regression (normality, linearity, homoscedasticity, and absence of multicollinearity) were verified through inspection of residual plots, variance inflation factors (VIF), and statistical tests. All assumptions were met. Predictor variables included the three Dark Triad traits, the three mindfulness facets, three alexithymia dimensions (N-DAF, P-DAF, EOT), and the control variables (age, gender). Gender was treated as a binary control variable (1 = male, 2 = female). Participants who identified as non-binary or preferred not to disclose were excluded from analyses involving this variable due to low subgroup size (*n* = 15). For significant relationships between Dark Triad traits and psychopathology identified in the regression analyses (controlling for other predictors), moderation analyses were conducted to test for interaction effects. Specifically, we examined whether the alexithymia facet N-DAF score (as a risk factor) and mindfulness facets (as protective factors) moderated these associations. Each moderation analysis was conducted using interaction terms created by centering the predictor (Dark Triad trait) and moderator (alexithymia or mindfulness facet) variables and computing their product. Moderation analyses followed Hayes’s (Hayes, 2017) Model 1 framework for simple moderation, in which each interaction term (e.g., Dark Triad trait × Alexithymia or Dark Triad trait × Mindfulness facet) was tested in a separate regression model. This approach allowed us to examine the unique moderating role of each construct while avoiding multicollinearity among multiple interaction terms. The centered main effects and interaction term were then entered into separate linear regression models predicting each psychopathology outcome (depression, anxiety, or stress). A significant interaction term (*p* < 0.05) was interpreted as evidence of moderation. Where significant interactions were found, simple slopes analyses were conducted at low (−1 *SD*), mean, and high (+1 *SD*) levels of the moderator using the *sim_slopes()* function, and the effects were visualized using interaction plots. The additional variance explained by each interaction term (Δ*R*^2^) was computed by comparing the main-effects model to the full model including the interaction, using an *F*-test for nested models. All correlation and regression coefficients are reported with exact *p*-values and standardized estimates, where applicable. Effect sizes were interpreted based on Cohen’s conventional benchmarks (Cohen, 2013). Alpha was set at 0.05. Multiple comparisons in correlational analyses were corrected for using the False Discovery Rate (FDR) procedure (Benjamini and Hochberg, 1995).

## 3 Results

### 3.1 Control variables

Control variables included age and gender, and their associations with all major study constructs (Dark Triad traits, psychopathology, alexithymia, and mindfulness) were systematically examined. For descriptive questionnaire information, see Table 1; all main study constructs are presented by gender in Appendix.

Gender differences were observed in several domains. Males reported significantly higher levels of narcissism (*z* = −3.60, *p* < 0.001, *r* = 0.15) and psychopathy (*z* = −3.42, *p* < 0.001, *r* = 0.14) than females, while no gender difference was found for Machiavellianism (*p* > 0.05). In terms of psychopathology, females reported significantly higher levels of anxiety (*z* = −3.66, *p* < 0.001, *r* = 0.15) and stress (*z* = −4.21, *p* < 0.001, *r* = 0.18), whereas no significant gender difference was found for depression (*p* > 0.05). Among the mindfulness facets, males scored significantly higher on non-reactivity to inner experience (*z* = −2.67, *p* = 0.008, *r* = 0.11), but no gender differences were found for acting with awareness or non-judging of inner experience (all *p* > 0.05). No significant gender differences were observed for alexithymia (N-DAF, P-DAF, EOT; all *p* > 0.05).

Age was negatively associated with several key variables. Specifically, older age was significantly correlated with lower levels of depression (*τ* = −0.08, *p* = 0.008), anxiety (*τ* = −0.18, *p* < 0.001), Machiavellianism (*τ* = −0.09, *p* = 0.002), and narcissism (*τ* = −0.16, *p* < 0.001), as well as with lower levels of all alexithymia facets (N-DAF: *τ* = −0.15, *p* < 0.001; P-DAF: *τ* = −0.13, *p* < 0.001; EOT: *τ* = −0.16, *p* < 0.001).

### 3.2 Kendall’s *τ* correlations

Bivariate Kendall’s *τ*-*b* correlations among the main variables are presented in Table 2.

**Table 2:** Correlations between Dark Triad traits, psychopathology, mindfulness and alexithymia measures.

	1	2	3	4	5	6	7	8	9.	10	11	12
1. DD-Mach		0.65***	0.60***	0.12***	0.13***	0.13***	−0.15***	−0.16***	0.09**	0.15***	0.18***	0.13***
2. DD-Narc			0.65***	0.17***	0.18***	0.13***	−0.14***	−0.17***	0.04	0.18***	0.23***	0.17***
3. DD-Psych				0.18***	0.17***	0.17***	−0.16***	−0.20***	0.03	0.16***	0.20***	0.14***
4. DASS-Dep					0.55***	0.60***	−0.26***	−0.40***	−0.10**	0.35***	0.30***	0.28***
5. DASS-An						0.60***	−0.26***	−0.37***	−0.07	0.34***	0.30***	0.26***
6. DASS-St							−0.27***	−0.34***	−0.11***	0.29***	0.22***	0.21***
7. ActAw								0.32***	−0.17***	−0.24***	−0.21***	−0.19***
8. Njud									0.00	−0.29***	−0.25***	−0.26***
9. Nreac										−0.05	0.01	−0.01
10. N-DAF											0.68***	0.69***
11. P-DAF												0.67***
12. EOT												

DD, Dirty Dozen, Mach, Machiavellianism. Narc, narcissism. Psych, psychopathy. DASS, Depression Anxiety Stress Scales. Dep, Depression. An, Anxiety. St, Stress. ActAw, acting with awareness. Njud, non-judging of inner experience. Nreac, non-reactivity to inner experience. G-DAF, General Difficulty Appraising Feelings. EOT, General Externally Oriented Thinking. *p < 0.05, **p < 0.01, ***p < 0.001. FDR-correction has been applied.

### 3.3 Regression analyses

To investigate whether Dark Triad traits explain unique variance in psychopathology beyond other psychological factors, three multiple regression analyses were conducted with depression, anxiety, and stress as dependent variables. Independent variables included the three Dark Triad traits (Machiavellianism, narcissism, psychopathy), three mindfulness facets (acting with awareness, non-judging of inner experience, non-reactivity to inner experience), and all three alexithymia dimensions (N-DAF, P-DAF, EOT). Age and gender were included as control variables. See Table 3 for an overview of the three regressions.

**Table 3:** Regression analyses predicting depression, anxiety and stress.

Dependent variable	Predictors	*B*	SE	95% CI *B*		*β*	*t*	*p*	*R*^2^
				Lower bound	Upper bound				
DASS_Dep	Age	−0.04	0.03	−0.10	0.03	−0.04	−1.17	0.244	0.40
	Gender	0.41	0.46	−0.49	1.31	0.03	0.89	0.374	
	**DD-Mach**	**−0.79**	**0.28**	**−1.34**	**−0.23**	**−0.17**	**−2.81**	**0.005**	
	DD-Narc	0.35	0.34	−0.32	1.03	0.07	1.03	0.302	
	DD-Psych	0.49	0.27	−0.03	1.01	0.12	1.85	0.065	
	**ActAw**	**−0.82**	**0.30**	**−1.39**	**−0.24**	**−0.11**	**−2.77**	**0.006**	
	**Njud**	**−2.10**	**0.23**	**−2.54**	**−1.66**	**−0.37**	**−9.32**	**<0.001**	
	**Nreac**	**−0.73**	**0.24**	**−1.21**	**−0.26**	**−0.11**	**−3.03**	**0.003**	
	**N-DAF**	**0.60**	**0.15**	**0.30**	**0.91**	**0.30**	**3.92**	**<0.001**	
	P-DAF	0.13	0.16	−0.19	0.44	0.06	0.79	0.432	
	EOT	−0.06	0.04	−0.13	0.14	−0.11	−1.56	0.118	
DASS_An	**Age**	**−0.06**	**0.03**	**−0.12**	**−0.01**	**−0.08**	**−2.20**	**0.028**	0.39
	**Gender**	**1.60**	**0.43**	**0.76**	**2.44**	**0.13**	**3.75**	**<0.001**	
	**DD-Mach**	**−0.56**	**0.26**	**−1.08**	**−0.05**	**−0.13**	**−2.15**	**0.032**	
	DD-Narc	0.49	0.32	−0.14	1.11	0.10	1.53	0.127	
	DD-Psych	0.28	0.25	−0.21	0.76	0.07	1.11	0.267	
	**ActAw**	**−0.96**	**0.27**	**−1.50**	**−0.43**	**−0.14**	**−3.51**	**<0.001**	
	**Njud**	**−1.75**	**0.21**	**−2.17**	**−1.34**	**−0.33**	**−8.35**	**<0.001**	
	Nreac	−0.37	0.22	−0.81	0.07	−0.06	−1.65	0.100	
	**N-DAF**	**0.60**	**0.14**	**0.31**	**0.88**	**0.32**	**4.15**	**<0.001**	
	P-DAF	0.21	0.15	−0.08	0.51	0.10	1.45	0.148	
	**EOT**	**−0.10**	**0.03**	**−0.16**	**−0.03**	**−0.21**	**−3.00**	**0.003**	
DASS_St	Age	<0.01	0.03	−0.06	0.06	0.00	−0.01	0.989	0.34
	**Gender**	**1.56**	**0.44**	**0.70**	**2.41**	**0.13**	**3.58**	**<0.001**	
	DD-Mach	−0.07	0.27	−0.59	0.45	−0.02	−0.26	0.792	
	DD-Narc	−0.46	0.32	−1.10	0.17	−0.10	−1.43	0.153	
	**DD-Psych**	**0.73**	**0.25**	**0.24**	**1.23**	**0.19**	**2.90**	**0.004**	
	**ActAw**	**−1.39**	**0.28**	**−1.94**	**−0.84**	**−0.21**	**−4.99**	**<0.001**	
	**Njud**	**−1.44**	**0.21**	**−1.86**	**−1.02**	**−0.28**	**−6.74**	**<0.001**	
	**Nreac**	**−1.00**	**0.23**	**−1.45**	**−0.55**	**−0.16**	**−4.37**	**<0.001**	
	**N-DAF**	**0.45**	**0.15**	**0.17**	**0.74**	**0.25**	**3.11**	**0.002**	
	P-DAF	−0.06	0.15	−0.36	0.23	−0.03	−0.43	0.670	
	EOT	−0.03	0.03	−0.09	0.04	−0.06	−0.79	0.431	

DD, Dirty Dozen; Mach, Machiavellianism; Narc, narcissism; Psych, psychopathy; DASS, Depression Anxiety Stress Scales; Dep, Depression; An, Anxiety; St, Stress; ActAw, acting with awareness; Njud, non-judging of inner experience; Nreac, non-reactivity to inner experience; N-DAF, Difficulty Appraising Negative Feelings; P-DAF, Difficulty Appraising Positive Feelings; EOT, General Externally Oriented Thinking. Significant predictors are marked in bold font.

The regression model predicting depression was statistically significant, *F*(10, 561) = 32.94, *p* < 0.001, accounting for 39.7% of the variance in depressive symptoms (*R^2^* = 0.397). Significant unique predictors in the model included Machiavellianism (negative predictor, *p* = 0.005), all three mindfulness facets (negative predictors, all *p* < 0.006), and N-DAF (positive predictor, *p* < 0.001).

The regression model predicting anxiety was statistically significant, *F*(10, 561) = 32.28, *p* < 0.001, accounting for 39.2% of the variance in anxiety symptoms (*R^2^* = 0.392). Among the predictors, age (negative predictor, *p* = 0.028), gender (positive predictor, *p* < 0.001), Machiavellianism (negative predictor, *p* = 0.032), acting with awareness (negative predictor, *p* < 0.001), non-judging of inner experience (negative predictor, *p* < 0.001), and both alexithymia facets N-DAF (positive predictor, *p* < 0.001) and EOT (negative predictor, *p* = 0.003) contributed significantly to the model.

The regression model predicting stress was also statistically significant, *F*(10, 561) = 26.21, *p* < 0.001, explaining 34.4% of the variance in perceived stress (*R^2^* = 0.344). Significant unique predictors included gender (positive predictor, *p* < 0.001), psychopathy (positive predictor, *p* = 0.004), all mindfulness facets (negative predictor, *p* < 0.001) and N-DAF (positive predictor, *p* = 0.002).

### 3.4 Moderation analyses

**Figure 1:**
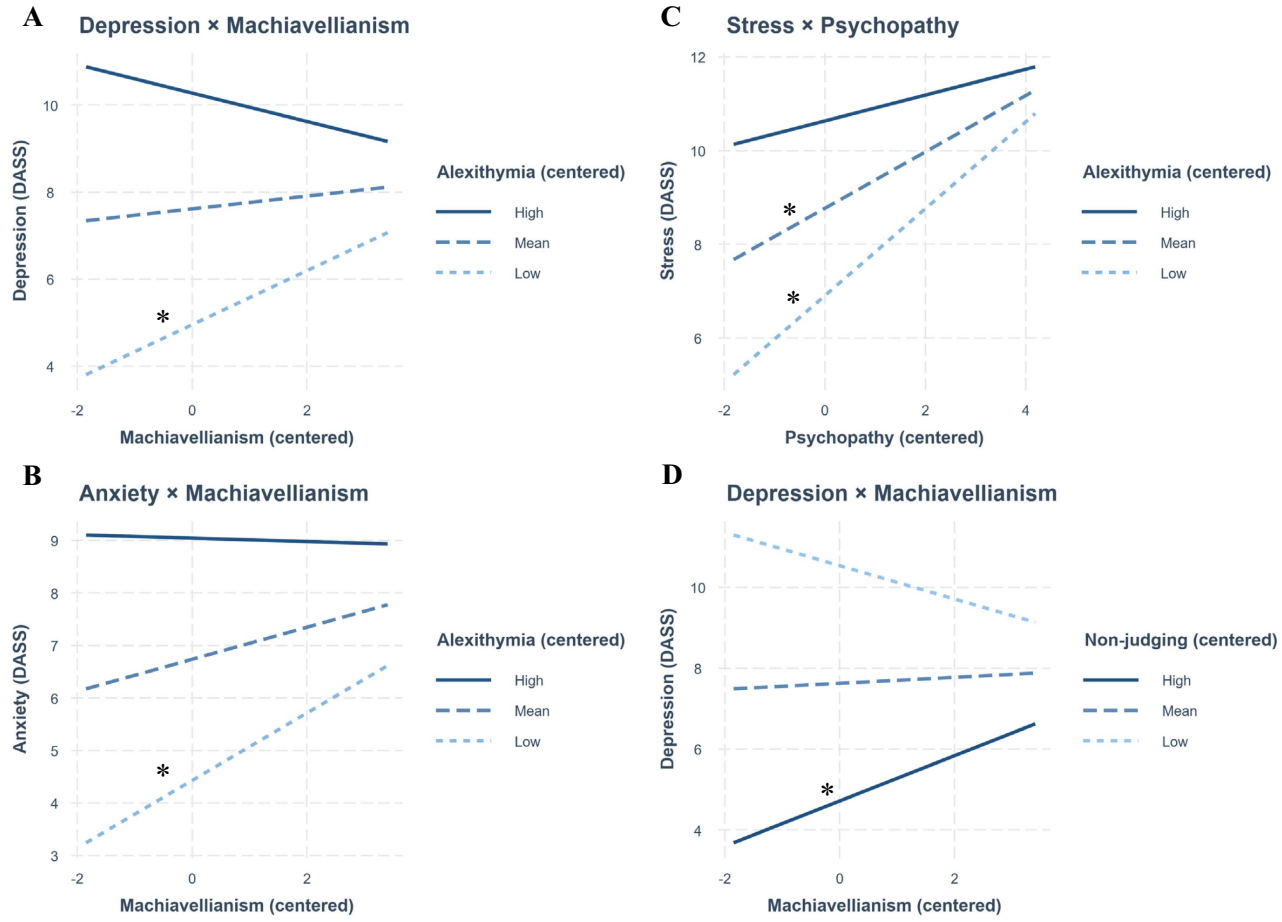
Significant moderation analyses of Dark Triad traits and psychopathology by alexithymia (Difficulty Appraising Negative Feelings) **(A–C)** and Mindfulness (non-judging of inner experience) **(D)**. Note that the negative interaction coefficient (Table 4) reflects a decreasing slope magnitude with increasing alexithymia. Asterisks indicate statistically significant slopes.

To follow up on the significant relationships between Dark Triad traits and psychopathology identified in the regression analyses, we conducted a series of moderation analyses to examine potential buffering or amplifying effects. Specifically, the alexithymia facet N-DAF (as a potential risk factor) and the three mindfulness facets (acting with awareness, non-judging of inner experience, non-reactivity to inner experience; as potential protective factors) were tested as moderators of these relationships. See Table 4 (alexithymia) and Table 5 (mindfulness) for an overview of interaction terms within the moderation analyses and Figure 1 for graphical depictions of significant moderation analyses.

**Table 4:** Moderation of the relationship between Dark Triad traits and psychopathology by alexithymia (N-DAF).

Dark Triad trait	Outcome	*b*	*SE*	*p*
DD-Mach	**DASS-Dep**	−0.17	0.06	**0.007**
	**DASS-An**	−0.12	0.06	**0.038**
DD-Psych	**DASS-St**	−0.12	0.05	**0.018**

N-DAF, Difficulty Appraising negative Feelings; DD, Dirty Dozen; Mach, Machiavellianism; Psych, psychopathy; DASS, Depression Anxiety Stress Scales; Dep, Depression; An, Anxiety; St, Stress. *p* < 0.05 are marked in bold font.

**Table 5:** Moderation of the relationship between Dark Triad traits and psychopathology by selected mindfulness facets.

Dark Triad trait	Outcome	ActAw			Njud			Nreac		
		*b*	*SE*	*p*	*b*	*SE*	*p*	*b*	*SE*	*p*
DD-Mach	**DASS-Dep**	0.51	0.26	0.054	0.50	0.17	**0.004**	0.15	0.25	0.553
	**DASS-An**	0.18	0.24	0.450	0.17	0.16	0.287	-	-	-
DD-Psych	**DASS-St**	−0.09	0.20	0.659	0.20	0.15	0.163	0.02	0.19	0.917

DD, Dirty Dozen; Mach, Machiavellianism; Narc, narcissism; Psych, psychopathy; DASS, Depression Anxiety Stress Scales; Dep, Depression; An, Anxiety; St, Stress; ActAw, acting with awareness; Njud, non-judging of inner experience; Nreac, non-reactivity to inner experience. *p* < 0.05 are marked in bold font.

Significant moderation effects were found for Machiavellianism and depressive symptoms: alexithymia moderated the relationship negatively, (*B* = −0.20, *p* = 0.007), while the mindfulness facet of non-judging of inner experience (*B* = 0.50, *p* = 0.004) positively moderated this relationship. Similarly, Machiavellianism’s association with anxiety was negatively moderated by alexithymia (*B* = −0.12, *p* = 0.038), though no mindfulness facets showed significant effects (all *p* > 0.05). For stress, psychopathy interacted significantly negative with alexithymia (*B* = −0.12, *p* = 0.018), but no mindfulness facet significantly moderated this relationship (*p* > 0.05).

For the Machiavellianism × Alexithymia interaction predicting depression, the slope of Machiavellianism was significant at low alexithymia (*B* = 0.62, *p* = 0.015) but nonsignificant at mean and high alexithymia (*B*s = 0.15 and −0.33, *p*s > 0.188), indicating attenuation of the effect at higher alexithymia. A similar pattern was found for anxiety (*B* = 0.64, *p* = 0.007 at low alexithymia; nonsignificant at mean and high alexithymia). For the Psychopathy × Alexithymia interaction predicting stress, the relationship was strongest at low (*B* = 0.93, *p* < 0.001) and mean (*B* = 0.60, *p* < 0.001) alexithymia, but weakened at high alexithymia (*B* = 0.28, *p* = 0.149). Finally, for the Machiavellianism × Non-judging interaction predicting depression, Machiavellianism was associated with higher depression only at high levels of non-judging (*B* = 0.56, *p* = 0.024), suggesting that greater non-judgment may buffer the negative impact of Machiavellianism.

The inclusion of interaction terms accounted for small but statistically significant increases in explained variance (Δ*R*^2^ = 0.006–0.010). Specifically, the Machiavellianism × Alexithymia interaction predicted additional variance in depressive symptoms [Δ*R*^2^ = 0.010, *F*(1, 559) = 7.37, *p* = 0.007] and anxiety [Δ*R*^2^ = 0.006, *F*(1, 559) = 4.34, *p* = 0.038]. Similarly, the Psychopathy × Alexithymia interaction explained a further 0.8% of variance in stress [Δ*R*^2^ = 0.008, *F*(1, 559) = 5.63, *p* = 0.018]. For depression, the interaction between Machiavellianism and the mindfulness facet non-judging of inner experience accounted for an additional 1.1% of variance in depressive symptoms [Δ*R*^2^ = 0.011, *F*(1, 559) = 8.55, *p* = 0.004]. Although these effect sizes were small (<1%), they are consistent with moderation effects typically observed in individual-differences research.

## 4 Discussion

In the present study, we aimed to investigate the relationships between Dark Triad traits and psychopathology, with a particular focus on alexithymia as a potential risk factor and mindfulness as a potential resilience factor. For this purpose, we analyzed data from a student sample which, despite its relative homogeneity in educational background, showed sufficient variability in both Dark Triad traits and psychopathological symptoms.

Our findings demonstrate that even within a student sample, there are robust, albeit small, associations between Dark Triad traits and various forms of psychopathology. Specifically, we found positive relationships with symptoms of anxiety, depression, and stress. These results align with previous research indicating that elevated levels of Machiavellianism, narcissism and/or psychopathy traits are generally associated with increased psychological vulnerability (Papageorgiou et al., 2023; Bonfá-Araujo et al., 2023; Gómez-Leal et al., 2019; Lyons et al., 2019; Birkás et al., 2016; Wei et al., 2025). Building on these findings, we further examined whether alexithymia and mindfulness also show associations with these traits in our sample.

Consistent with prior research (Jonason and Krause, 2013; Wastell and Booth, 2003; Sheikhi et al., 2017; Schimmenti et al., 2019; Di Tella et al., 2024; Burghart and Mier, 2022), we observed small but significant positive correlations between alexithymia and Dark Triad traits. Specifically, greater difficulties in appraising emotions (i.e., difficulty identifying and describing positive or negative emotions) were linked to higher levels of Dark Triad characteristics, alongside less attention to emotions (i.e., a more Externally Oriented Thinking style). Alexithymia is known to impair emotion regulation abilities, as difficulties in recognizing and interpreting emotions can hinder effective emotional decision making (Preece et al., 2023). This impairment may, in turn, intensify impulsive tendencies associated with Dark Triad traits. Introspection about one’s own emotions can also be useful for facilitating understanding of and empathy towards others’ emotions (Brett et al., 2023), which may be of less interest to those high in Dark Triad traits, or alternatively alexithymia may contribute to unempathetic dispositions.

The mindfulness facets acting with awareness and non-judging of inner experience showed small yet robust negative associations with all three Dark Triad traits. This suggests that individuals with a more mindful disposition tend to report lower levels of these socially aversive personality traits. This contrasts with some of the previous findings in the field. One possible reason for this discrepancy may lie in methodological differences. In a study by Scavone (2017), mindfulness was operationalized and analyzed with an additional facet—describing—which substantially overlaps with the construct of alexithymia and was therefore excluded from our analyses. While ‘describing’ refers to the mindful ability to label internal experiences with words, alexithymia represents a deficit in exactly this capacity. This conceptual overlap—despite their opposing directions—likely explains the reversed scoring patterns (i.e., higher values indicating strength in ‘describing’ vs. difficulty in alexithymia). A second study, unfortunately, did not offer any explanation or theoretical interpretation as to why a positive association between mindfulness and psychopathy was found, nor why no significant relationships were observed for narcissism or Machiavellianism (Aldbyani and Al-Abyadh, 2022). Their results appear somewhat counterintuitive from a conceptual perspective, given that traits such as impulsivity and emotional dysregulation—typically associated with psychopathy—stand in contrast to the core principles of mindfulness. However, inconsistent results across studies highlight the complexity of these relationships and the need for further theoretical integration. In our study, the observed negative associations between mindfulness and Dark Triad traits align well with our theoretical reasoning: mindfulness as a non-judgmental awareness of one’s internal experience, stands in opposition to the self-deceptive tendencies and low self-awareness characteristic of narcissism and Machiavellianism. Similarly, the emotional detachment and lack of empathy associated with psychopathy and Machiavellianism may reflect an absence of mindful engagement with others. Thus, our findings contribute to a more nuanced understanding of the relationship between Dark Triad traits and mindfulness. However, the current state of research remains mixed, and further studies with well-powered and gender-balanced samples are needed to draw more definitive conclusions.

To further examine these associations, we conducted separate regression analyses for depression, anxiety, and stress, including mindfulness, alexithymia, and the Dark Triad traits as predictors, while controlling for age and gender. This approach allowed us to explore the relative contributions of these constructs to psychological distress beyond simple bivariate relationships.

The results revealed distinct patterns across the three models, each explaining a substantial proportion of variance in psychological distress (34–40%), highlighting the relevance of the included predictors. Machiavellianism was negatively associated with depression and anxiety—an intriguing finding, given its previously observed positive correlations both in our data and in prior research (Wei et al., 2025). This reversal of direction in the regression analyses indicates that when all predictors were considered simultaneously, the unique contribution of Machiavellianism emerged as negative rather than positive. Such a pattern is consistent with a potential multivariate suppressor effect, where shared variance with related constructs (e.g., mindfulness or alexithymia) is partialed out. This suggests that when accounting for related vulnerability factors such as low mindfulness or high alexithymia regarding negative emotions, Machiavellianism may not necessarily be linked to psychological burden. On the contrary, its negative association with internalizing symptoms might reflect adaptive components of the trait, such as emotional detachment, cognitive control, or strategic coping styles that buffer against distress under certain conditions (Mehta et al., 2025). In contrast, psychopathy emerged as a significant positive predictor of stress, aligning with both our correlational findings and existing literature. This supports the view that psychopathic traits—marked by impulsivity, emotional dysregulation, and interpersonal antagonism—contribute to increased vulnerability to high-arousal states like stress, even when other risk and resilience factors are considered. Mindfulness showed consistent negative associations with all three domains of psychological distress, underscoring its robust protective role and echoing prior evidence of its buffering effects. Conversely, alexithymia was positively associated with psychopathology, supporting the notion that difficulties in identifying and expressing negative emotions—but not positive ones—heighten vulnerability to internalizing symptoms (Preece et al., 2024). Some differential patterns emerged particularly in the anxiety model: while the mindfulness facet of non-reactivity to inner experience did not significantly predict anxiety levels, the alexithymia facet EOT emerged as a significant negative predictor—alongside N-DAF, which was a consistent positive predictor across all three models. This finding regarding EOT is noteworthy, as both mindfulness and alexithymia are conceptually relevant in the context of anxiety symptoms. Anxiety, as a rapid, evolutionarily conserved emotional response, may be particularly difficult to reconcile with the mindfulness-based capacity to let thoughts and emotions arise and pass without judgment or entanglement (Baer et al., 2006). In contrast, the negative association of EOT with anxiety may reflect a conceptually similar mechanism from the opposite perspective, namely a heightened inward focus in anxious individuals rather than an outward, externally oriented attentional style [see also, Winterstein et al. (2024)]. This suggests that not all facets of these constructs are equally relevant across different types of distress which may have contributed to the mixed findings in the literature, depending on methodology. In general, difficulties appraising negative feelings clearly appear to be relevant in all three psychopathology areas, whereas difficulties appraising positive emotions show no associations—highlighting the importance of differentiating emotional valence (positive vs. negative) in understanding these relationships.

The significant effects of gender and age, especially in the anxiety and stress models, highlight the ongoing relevance of sociodemographic variables in mental health research. Women reported higher levels of anxiety and stress, which may reflect greater psychosocial burden or differing coping strategies; however, previous research among university students in different countries has shown mixed patterns, and our findings contribute to this heterogeneous evidence base (Li et al., 2021; Pang et al., 2021). The fact that we did not find a gender effect in depression may be due to the high proportion of women in our sample. In contrast, older individuals tended to report lower levels of anxiety and stress, possibly due to improved emotional regulation or age-related shifts in perspective and life circumstances, a pattern that has also been observed across all DASS scales in previous research (Varma et al., 2021). The significant effects of gender and age, especially in the anxiety and stress models, highlight the ongoing relevance of sociodemographic variables in mental health research.

Building on these findings, we further investigated whether mindfulness and alexithymia moderate the relationships between Dark Triad traits and psychopathology. The mindfulness facet of non-judging of inner experience moderated the relationship between Machiavellianism and depressive symptoms. A closer visual inspection of the interaction suggests a more complex pattern: at low levels of mindfulness facet non-judging of inner experience, Machiavellianism was negatively associated with depression, suggesting that a critical or evaluative stance toward one’s internal states may buffer against depressive symptoms in individuals high in Machiavellian traits. At moderate levels of mindfulness, the association between Machiavellianism and depression became almost negligible. However, at high levels of mindfulness, the relationship reversed—Machiavellianism was positively associated with depressive symptoms. Statistical testing indicated that only the slope at high levels of non-judging was significant. This pattern suggests a complex interaction, in which the typically protective attitude of non-judgment may, in the context of high Machiavellian traits, lose its buffering effect or even exacerbate emotional distress. One possible explanation is that for individuals with high Machiavellianism, a non-judgmental stance might lead to increased self-awareness without the corresponding capacity for emotional integration, thereby heightening psychological burden. These findings highlight that mindfulness is not universally protective and underscore the importance of tailoring interventions to individual personality profiles. Beyond mindfulness, future research should also examine alternative protective factors that may be particularly relevant for individuals with higher levels of dark personality traits. For example, self-compassion—which is linked to mindfulness (Neff et al., 2020) and can buffer against psychological distress (Muris and Otgaar, 2023) also provide similar benefits in these populations.

Difficulties in appraising negative feelings—the most consistent alexithymia facet—moderated the relationship between Machiavellianism and both depression and anxiety, although the pattern appears more complex than a simple linear association. Notably, the direction of this association varied depending on the level of alexithymia: while individuals with low levels of alexithymia visually showed a positive association between Machiavellianism and depressive/anxious symptoms, this relationship weakened in a median level of alexithymia and reversed at higher levels of alexithymia. Statistical testing indicated that only the slope at low alexithymia was significant, while slopes at median and high levels were not statistically significant. This trend suggests a complex, non-linear moderating effect, whereby the psychological impact of Machiavellianism on mental health may differ substantially depending on an individual’s ability to identify and describe their negative emotions. One possible explanation for this moderation effect is that higher levels of alexithymia may act as a psychological buffer: when negative emotions such as guilt, shame, or anxiety are not consciously recognized, the distress typically associated with maladaptive traits may be diminished or suppressed. In this sense, emotional unawareness regarding negative feelings could serve a protective or functional role, particularly in traits like Machiavellianism, by shielding the individual from emotional discomfort. However, this pattern may not generalize across all Dark Triad traits. For example, in the case of psychopathy, alexithymia was associated with a stronger link to stress, suggesting that emotional unawareness regarding negative emotions may, in other cases, exacerbate rather than mitigate psychological strain. Notably, the pattern suggests that individuals with higher psychopathy may experience elevated stress when they retain some emotional awareness, while high alexithymia may buffer perceived stress to some extent. This finding is particularly notable for psychopathy, as individuals high in psychopathic traits already tend to exhibit emotional detachment and low empathy, which may interact with alexithymia in shaping stress responses. Statistical testing indicated that this association was significant at low and medium levels of alexithymia, but not at high levels. Indeed, outside of the Dark Triad, high alexithymia is most typically associated with poorer mental health outcomes (Preece and Sikka, 2024).

These findings suggest that the interplay between Machiavellianism and alexithymia is highly nuanced. It may be that individuals high in Machiavellianism represent a particularly challenging group: mindfulness does not appear to buffer symptoms, alexithymia becomes less predictive, and yet psychopathological symptoms remain elevated. This could reflect a population that is especially difficult to engage in therapeutic work due to limited emotional insight and low motivation for change. Overall, alexithymia regarding negative feelings emerged as a more relevant and consistent moderator than mindfulness, particularly in the context of psychopathy, but also in more complex ways for Machiavellianism.

### 4.1 Limitations

Several limitations should be acknowledged when interpreting the findings of this study. First, the sample consisted primarily of university students, with a notable overrepresentation of female participants, which may limit the generalizability of the results. Moreover, the findings are based on a specific cultural and social context, which may further constrain their applicability to other populations. Future research should examine whether the observed relationships between dark personality traits, protective factors such as mindfulness or self-compassion, and psychological outcomes hold across diverse cultural and social environments. Second, all constructs were measured using self-report questionnaires, which are inherently subject to biases such as social desirability and limited self-awareness. This is particularly relevant in the context of dark personality traits, where individuals may consciously or unconsciously underreport certain tendencies. However, the online format—without face-to-face interaction or observation—may have reduced socially desirable responding and thereby enhanced data validity. Supporting this assumption, the full range of responses was utilized for narcissism and psychopathy, and nearly the full range (up to 6.25 out of a maximum of 7 points) for Machiavellianism. Third, the present study assessed only *grandiose* narcissism as part of the Dark Triad. Thus, potential effects associated with *vulnerable* narcissism may not have been detected, although this focus aligns with the Dark Triad framework. Lastly, the study’s cross-sectional design precludes any inference of causal relationships. While the moderation patterns observed are theoretically meaningful, they remain correlational. Longitudinal research is needed to clarify the directionality and temporal dynamics of these associations.

### 4.2 Strengths

Despite these limitations, the present study offers several notable strengths. First, it provides a novel integration of conceptually relevant variables, combining dark personality traits, alexithymia, with a focus on valence differentiation, and mindfulness to explore psychological distress. The inclusion of moderation analyses allowed for a more fine-grained understanding of how these traits interact to influence symptoms of depression, anxiety, and stress. Furthermore, by examining both risk (alexithymia) and potential protective factor (mindfulness), the study adopts a balanced perspective that may inform future intervention efforts. Methodologically, the use of well-validated instruments enhances the reliability and validity of the findings. The study also benefits from a large and heterogeneous sample, including a substantial proportion (nearly 40%) of participants with self-reported mental health conditions, which enhances the ecological validity and generalizability of the results. Moreover, the dimensional assessment of psychopathology, rather than relying on diagnostic categories, allowed for the capture of both clinical and subclinical symptom levels across a broader range of participants.

### 4.3 Conclusion

In summary, the present study confirmed that Dark Triad traits are positively associated with psychopathological symptoms, including depression, anxiety, and stress. However, when considering potential moderators such as alexithymia and mindfulness, a more nuanced picture emerged. The association between psychopathy and stress was strengthened by higher levels of alexithymia, highlighting the role of emotional unawareness of negative emotions as a psychological risk factor. Narcissism initially showed correlations with psychological distress but no longer demonstrated significant associations once additional or moderating factors were considered. In contrast, for Machiavellianism, both alexithymia and the mindfulness facet of non-judging of inner experience functioned as either risk or resilience factors, depending on their levels—alexithymia (difficulties appraising negative feelings) in relation to both depressive and anxiety symptoms, and non-judging specifically in relation to depressive symptoms. These findings underscore the importance of context-sensitive approaches when examining personality and psychopathology. This complexity also points to potential therapeutic challenges. Individuals high in Machiavellianism may respond differently—or even adversely—to interventions based on mindfulness or emotional awareness towards negative feelings. Therefore, further research is warranted to better understand these interactions and to develop individualized clinical strategies for individuals with pronounced dark personality traits.

## Data Availability

The raw data supporting the conclusions of this article will be made available by the authors, without undue reservation.

## Footnotes

1 Subsets of this broader data-set have been used previously in other published papers (Johnston et al., 2025; Preece et al., 2023; Preece and Gross, 2023; Preece and Gross, 2024; Stulcbauer et al., 2024; Winterstein et al., 2024), but they examined different research questions.

## Appendix

**Table A1:** Descriptive statistics by gender.

Questionnaire measures	Male		Female	
	*M (SD)*	Range	*M* (*SD*)	Range
Machiavellianism (DD)	3.00 (1.20)	1.00–6.00	2.81 (1.15)	1.00–6.25
Narcissism (DD)	2.87 (1.08)	1.00–6.00	2.53 (1.06)	1.00–7.00
Psychopathy (DD)	3.16 (1.33)	1.00–6.00	2.72 (1.28)	1.00–7.00
Depression (DASS-21)	6.86 (5.27)	0.00–21.00	7.59 (5.63)	0.00–21.00
Anxiety (DASS-21)	5.20 (4.68)	0.00–21.00	7.06 (5.22)	0.00–21.00
Stress (DASS-21)	7.12 (5.07)	0.00–21.00	9.06 (4.97)	0.00–21.00
Externally Oriented Thinking (PAQ)	3.31 (1.40)	1.00–7.00	3.33 (1.36)	1.00–7.00
Difficulty Appraising Feelings (PAQ)	3.02 (1.33)	1.00–6.94	3.06 (1.25)	1.00–6.88
Total Alexithymia (PAQ)	74.83 (31.85)	24.00–167.00	75.66 (29.89)	24.00–166.00
Acting with awareness (FFMQ)	2.98 (0.78)	1.33–5.00	2.93 (0.74)	1.00–5.00
Non-judging of inner experience (FFMQ)	3.17 (0.90)	1.00–5.00	3.16 (1.00)	1.00–5.00
Non-reactivity to inner experience (FFMQ)	3.15 (0.80)	1.33–5.00	2.91 (0.82)	1.00–5.00

DD, Dirty Dozen; DASS-21, Depression Anxiety Stress Scales; FFMQ, Five Facet Mindfulness Questionnaire; PAQ, Perth Alexithymia Questionnaire.
